# Competing Manuscripts Based on the Same Study

**DOI:** 10.1590/S1516-31802000000100008

**Published:** 2000-01-02

**Authors:** International Committee of Medical Journal Editors

Editors may receive manuscripts from different authors offering competing interpretations of the same study. They have to decide whether to review competing manuscripts submitted to them more or less simultaneously by different groups or authors, or they may be asked to consider one such manuscript while a competing manuscript has been or will be submitted to another journal. Setting aside the unresolved question of ownership of data, we discuss here what editors ought to do when confronted with the submission of competing manuscripts based on the same study.

Two kinds of multiple submissions are considered: submissions by coworkers who disagree on the analysis and interpretation of their study, and submissions by coworkers who disagree on what the facts are and which data should be reported.

The following general observations may help editors and others dealing with this problem.

## Differences in Analysis or Interpretation

Journals would not normally wish to publish separate articles by contending members of a research team who have differing analyses and interpretations of the data, and submission of such manuscripts should be discouraged. If coworkers cannot resolve their differences in interpretation before submitting a manuscript, they should consider submitting one manuscript containing multiple interpretations and calling their dispute to the attention of the editor so that reviewers can focus on the problem. One of the important functions of peer review is to evaluate the authors’ analysis and interpretation and to suggest appropriate changes to the conclusions before publication. Alternatively, after the disputed version is published, editors may wish to consider a letter to the editor or a second manuscript from the dissenting authors. Multiple submissions present editors with a dilemma. Publication of contending manuscripts to air authors’ disputes may waste journal space and confuse readers. On the other hand, if editors knowingly publish a manuscript written by only some of the collaborating team, they could be denying the rest of the team their legitimate coauthorship rights.

## Differences in Reported Methods or Results

Workers sometimes differ in their opinions about what was actually done or observed and which data ought to be reported. Peer review cannot be expected to resolve this problem. Editors should decline further consideration of such multiple submissions until the problem is settled. Furthermore, if there are allegations of dishonesty or fraud, editors should inform the appropriate authorities.

The cases described above should be distinguished from instances in which independent, non-collaborating authors submit separate manuscripts based on different analyses of data that are publicly available. In this circumstance, editorial consideration of multiple submissions may be justified, and there may even be a good reason for publishing more than one manuscript because different analytical approaches may be complementary and equally valid.

